# Recommendations, Practices and Infrastructural Model for the Dental Radiology Set-up in Clinical and Academic Institutions in the COVID-19 Era

**DOI:** 10.3390/biology9100334

**Published:** 2020-10-13

**Authors:** Anu Sushanth. A, Kumar Chandan Srivastava, Deepti Shrivastava, Hala A. Hosni, Zafar Ali Khan, Khalid Al-Johani, Ibrahim A Alzoubi, Sasirekha B, Mohammed Ghazi Sghaireen, Mohammad Khursheed Alam

**Affiliations:** 1 Department of Oral Medicine & Radiology, Educare Institute of Dental Sciences, Malappuram, Kerala 676504, India; a.sushanth86@gmail.com; 2 Oral Medicine & Radiology, Department of Oral and Maxillofacial Surgery & Diagnostic Sciences, College of Dentistry, Jouf University, Sakaka 72345, Saudi Arabia; 3 Periodontics, Department of Preventive Dentistry, College of Dentistry, Jouf University, Sakaka 72345, Saudi Arabia; sdeepti20@gmail.com (D.S.); dr.ibrahim.alzoubi@jodent.org (I.A.A.); 4 Department of Oral and Maxillofacial Surgery & Diagnostic Sciences, College of Dentistry, Jouf University, Sakaka 72345, Saudi Arabia; dr.hala.hosni@jodent.org (H.A.H.); dr.zafar.khan@jodent.org (Z.A.K.); 5 Department of Oral diagnostic sciences, Faculty of Dentistry- King Abdulaziz University, Jeddah 80200, Saudi Arabia; kauoralmed@gmail.com; 6 Department Oral Medicine & Radiology, JKKN Dental College & Hospital, Komarapalayam 638183, India; sasipoorni@gmail.com; 7 Prosthodontics, Prosthetic Dental Sciences, College of Dentistry, Jouf University, Sakaka 72345, Saudi Arabia; dr.mohammed.sghaireen@jodent.org; 8 Orthodontics, Department of Preventive Dentistry, College of Dentistry, Jouf University, Sakaka 72345, Saudi Arabia; dr.mohammad.alam@jodent.org

**Keywords:** COVID-19, SARS COV-2, pandemic, dental radiography, infection control, infrastructural model, health care worker, physical distancing, decontamination, coronavirus prevention

## Abstract

**Simple Summary:**

Since the inception of COVID-19, the international and national agencies across the globe have issued several guidelines and recommendations for the public as well as to the healthcare professionals. The fast spreading nature of this disease has brought various changes in the screening and treatment protocols in medicine and dentistry. Oral radiology is an integral part of oral screening and various dental treatment strategies. During oral radiology procedures, oral radiologists/radiology technicians come in direct contact with the patient as well as their body fluids such as saliva. It increases the risk of disease transmission from patients to oral radiologist and vice versa. Hence, proper infection control protocol should be implemented with new guidelines. This paper explains the practices to be followed in the oral radiology clinics at various stages whether in commercial set up or academic institute. Additionally, it explains the various precautions and new recommendations to be taken during various radiographic techniques. To add more, emphasis the importance of safe distancing, barrier protection and decontamination to be followed in dental clinics to prevent the spread of infection. Furthermore, the feasibility and procedure of online teaching methods for oral radiology clinics has been discussed.

**Abstract:**

The pandemic of Coronavirus disease (COVID-19) has emerged as a global catastrophe that is plaguing mankind. In the past eight months since the world discovered about COVID-19, we learned a lot about server acute respiratory syndrome coronavirus 2 (SARS CoV-2) and perhaps there is much more to discover and understand about the virus. With the current understanding of the disease, we assume it will remain in an active state of transmission and progression among the community for a long time. Thus, it is advisable to adopt the disease’s prevention protocol in our daily and work routine. During this pandemic patient requiring dental treatment cannot be neglected and the role of dental imaging is crucial in delivering treatment. Hence, this article attempts to provide an evidence-based compilation about the mode of transmission and clinical features of COVID-19. It also throws light on the potential source of disease transmission in the dental radiology setting. In addition, it suggests preventive measures to curb the infection and infrastructural model of the clinical setting that will assist in achieving control over the disease transmission. This article intends to project a strategy about protocols, infrastructure, and daily activities in a dental radiology office that institutions can adopt with modifications according to their local scenario.

## 1. Introduction

On 31 December 2019, the China office of World health organization (WHO) reported about the cases of ‘viral pneumonia’ in Wuhan, People’s Republic of China [1] Shortly, on 9 January 2020 Chinese researchers have identified the pathogen for this atypical pneumonia like cases as novel Coronavirus [1,2]. This corona virus belongs to the beta-corona virus family which was initially given the name of 2019 novel coronavirus (2019-nCoV) [3]. After a considerable brainstorming and research this was renamed as severe acute respiratory syndrome coronavirus 2 (SARS-CoV-2) [4]. The disease caused by it was given the name of coronavirus disease 2019 (COVID-19) by WHO on 11 February 2020 [5]. Since then the cases of COVID-19 has been increasing tremendously around the world and WHO has recognized it as pandemic on 11 March 2020 [6]. This (SARS-CoV-2) has a potential to be transmitted rapidly from human to human. Symptoms such as fever, cough, shortness of the breath and diarrhoea are manifested in most of the diagnosed cases [7,8].

COVID-19 era has brought a significant change in the daily routine of every individual. The world is seeing an economic crisis along with enormous death causalities. Collectively with the efforts of healthcare workers (HCWs), public and government agencies, its spread can be minimised. New guidelines and recommendations for infection control protocols need to be implemented with outmost priority. Similar to medical set-up, the role of oral imaging in dental set-up is indispensable in diagnosis and treatment of elective as well as emergency cases. Among various possible routes of transmission of COVID-19, saliva seems to be a common factor between disease transmission and oral imaging [9]. Hence oral radiology offices can be a nidus for the spread of infection if strict precautions are not considered. For the safety of radiology staff and patients it is recommended to revise screening and clinical protocols of procedures [10].

Since COVID-19 is a new pandemic affecting world-wide, there are a lot of questions which needs to be addressed only through scientific resources [11]. There are many uncertainties related to COVID-19 such as the patient’s carrier status in pre-symptomatic or asymptomatic stage [12], effectiveness of the potential vaccine and stretch of this pandemic. Considering the gravity of COVID-19 and its potential to spread in the dental radiology office or institution, an attempt has been made to through light on how to combat COVID-19 in oral radiology clinics by modifying the layout. Additionally, this paper will highlight aspects to modify and implement new protocols in the oral radiology clinics both in academics and commercials set-up.

## 2. Mode of Transmission and Clinical Presentation

The primary mode of transmission for COVID-19 is through human to human either direct or contact [13]. Respiratory droplets seem to be the main source for transmission of infection [14]. Although, few studies have anticipated the possibility of faecal oral route as another mode of disease transmission [15].

In the oral cavity, saliva, oral epithelium, and salivary glands are potential routes of disease transmission [16]. It is believed that the oral epithelial cells contain surplus amount of angiotensin-converting enzyme 2 (ACE-2) receptor which helps the SARS CoV-2 to enter into the host cells [17]. SARS CoV-2 can enter the oral cavity by three different routes. Firstly, it can reach through the respiratory tract via liquid droplets. Secondly, it can enter from the blood stream via gingival crevicular fluid and thirdly, saliva from the infected salivary gland can reach to the oral cavity via salivary ducts [18]. Since salivary glands contain more ACE2 receptors than lungs, it is believed to be a potential reservoir of SARS CoV-2 leading to asymptomatic state [18].

In dentistry, aerosol generating procedures are of significant concern due to their high risk transmissibility [19]. From the recent studies it has been observed that virus has a potential to remain in air for longer duration and can even travel ˃6 feet [20]. These infected aerosols can remain suspended in air before they settle on the environmental surfaces. These surfaces in the dental operatory can be a potential source of disease transmission [13]. It has been reported that SARS CoV-2 virus can remain viable on various inanimate objects such as metal, wood, plastic and cardboard can range from 4 to 72 h [21].

The knowledge of HCWs about the clinical presentation in COVID-19 is considerably important in dentistry. These clinical presentation will help in telephone triaging, screening triage and teleradiology. The time taken for the initial symptoms to appear from the day of contamination with the virus generally ranges from one to fourteen days, with a mean of about 5 days [22]. The patient may be asymptomatic to mild symptomatic or severe symptoms progressing to acute respiratory distress syndrome to multiorgan failure. Most common manifestation includes fever, respiratory symptoms (shortness of breath, cough and dyspnoea) and gastrointestinal symptoms including diarrhoea and vomiting [23]. Recently dysgeusia and anosmia are also reported in many cases of COVID 19 patients [21].

## 3. Dental Radiology and COVID-19

In the background of COVID-19 pandemic, invariably every sector around the globe was forced to make changes in their work pattern and working environment. These changes are inevitable in order to return to work after months’ of lockdown. The nature and extent of changes depend on the amount of risk for disease transmission involved in a work set-up [24]. HCWs have always been in the forefront battling the pandemic, and thus also remained at the high risk for acquiring and transmitting disease [12,25,26]. Institutions involved in any form of health care delivery (academic, clinical care or both) required to maintain high standard of infection control protocol [27]. Dental radiology offices are no exception, and thus various recommendations and guidelines have been put forth in the lieu of COVID-19. The following section deals with the current update on recommendations applicable to dental radiology offices.

### 3.1. Source of Risks in Dental Radiology Office

Invariably all dental procedures involve a close contact with the patient for a reasonably long duration and thus bear an increased potential for cross contamination. Aerosol producing dental procedures are considered to have high risk for disease transmission, but fortunately dental radiology set-up barely includes any such procedures [28]. However, most dental radiology set-up is air conditioned with closed ventilation (no windows). Hence, inadvertently puts the HCWs in dental radiology units along with patients at risk [10]. The reflex of cough or sneeze produces aerosols which will stay suspended in air of enclosed offices/clinics for a longer duration [14]. Researchers have identified the probability of < 5 microns (aerosol particles) can readily penetrate the airway up till the alveolar spaces [29]. Hence the above-mentioned issues need to be considered in the preventive protocols in order to eliminate the disease progression.

### 3.2. Proposed Infrastructural Model/Layout for Dental Radiology Set-Up

**Figure 1:**
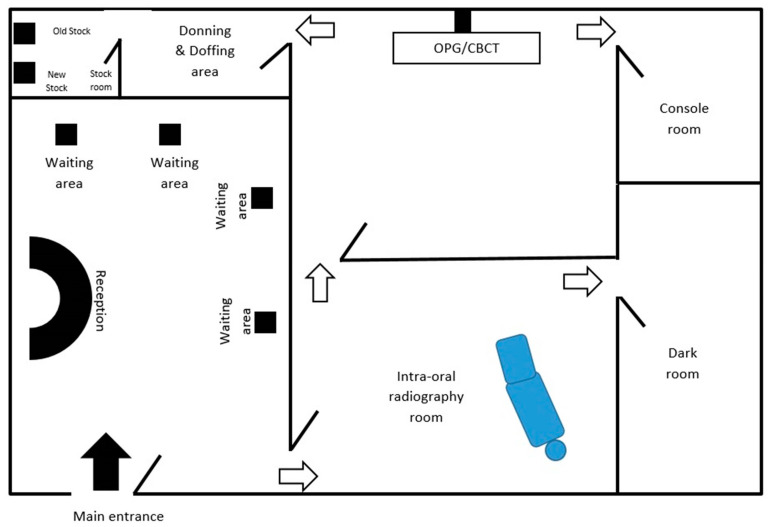
Layout of dental radiology office to ensure adherence to infection control protocol.

The radiation exposure to the community is generally the primary concern while designing a dental radiology set-up. Thickness of wall, incorporation of lead especially while constructing the surroundings of the operatory rooms and provision of lead barriers are some of the measures taken in the context of minimising radiation exposure. In this pandemic, considering the significance of infection control, the internal layout and ambiance needs some modification. In the waiting area, the sitting arrangement should be made in such a way so that a physical distance of 2 m can be maintained [3,8]. For maintaining hand hygiene, automatic or foot-controlled hand sanitizer dispenser should be stationed at key places. To reinforce the norms of distancing, etiquette to be displayed while speaking and coughing and hand hygiene, placards with easy description of the measures should be placed at multiple, strategic places [10]. Enclosed boxes of disposable masks and gloves should be kept in the waiting area and at the entrance of operatory room. To avoid crowding, prior appointment for radiological investigation can be given. Separate rooms for each imaging modality and its respective console unit are mandatory [9]. In case of multiple digital workstations, distancing of 1 m should be maintained. Isolated room for wearing and uncovering the PPEs is essential. The tactical placement of the old and new stock should be done so that new stock remains undisturbed till the potential virus remains viable on inanimate objects (Figure 1). To further setup the mitigation measures of cross-contamination, layout of the health centre can be modified to have separate entry and exit gates along with the placement of horizontal directional signage at multiple strategic place [30].

### 3.3. General Recommendations, Practices and Protocols in Oral Radiology Clinics

Strict hand hygiene practice always remains a mainstay in the prevention of the disease transmission and it holds true for dental radiology offices as well. Alcohol based disinfectants (70%) preferably in gel form are recommended to be used for at least 20 s [31]. Before entering the clinical area, the patient should undergo a screening triage which should include a temperature check and questions evaluating the risk status of the patient [9]. Recommendations suggest cut-off temperature of 37.3°C and patients above the cut-off are considered suspected for COVID-19. In such a situation, patient should be advised to undergo screening test for COVID-19 and should remain in quarantine for about 14 days [25]. Additionally, the level of oxygen saturation can be assessed with pulse oximeter. This will assist in identifying patient with possible silent hypoxia [32]. In a scenario, wherein a patient has a positive contact history in the recent past (14 days), then it is advisable to undergo confirmatory test for COVID-19 [25].

### 3.4. Specific Recommendations, Practices and Protocols in Oral Radiology Clinics

Considering the infective nature of COVID-19 with rapid transmission through close human contact, strict adherence to infection control measures seems crucial. In order to have better understanding the infection control protocols are described in the given below sections.

**Figure 2:**
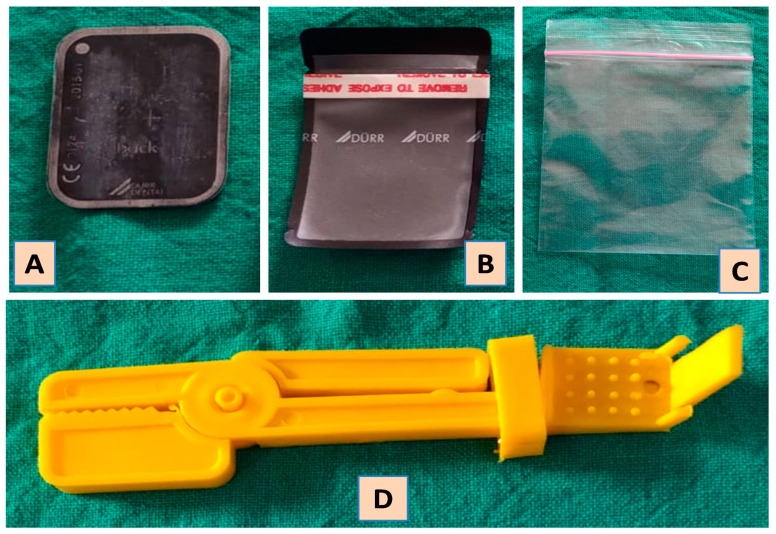
Intra oral radiography films and accessories. (**A**) Phosphor Storage Plates (Sensor); (**B**) Disposable sleeve for the sensor; (**C**) Outer (double barrier) transparent disposable sleeve for the sensor; (**D**) Eezee Grip/Snap A Ray Film holder for intra oral radiography.

**Figure 3:**
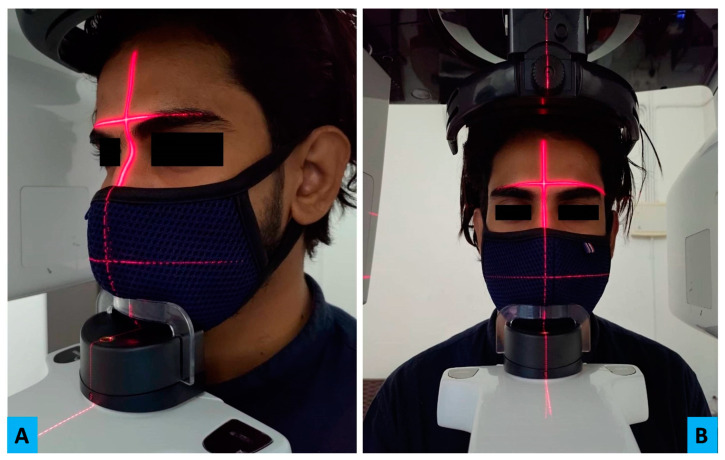
This figure depicts a patient’s lateral (**A**) and front (**B**) profile while being prepared for cone beam computed tomography (CBCT) imaging. Here, patient is shown wearing the face mask as a part of “new” infection control protocol. The red colour positioning lights can be seen assisting in the positioning of patient in the correct Sagittal and coronal plane.

- **Formulating and sustaining an infection control measures:** To ensure safety of patient’s as well dental office personal, a well-delineated infection control protocol is mandatory. Centre for disease control (CDC) guidelines for infection control protocols in dental radiology were given in 2003 [33]. The protocols need to be frequently reviewed and updated with emergence of new evidences [10]. Every HCW should be aware and committed towards its implementation in the routine functioning of the dental radiology office.
- **Selection of case:** Highly infective nature of SARS CoV-2 made the global health organization to issue recommendation, wherein initially only emergent dental treatments were carried out [25]. Later when ease in restrictions was applied, even elective treatment was allowed following strict infection control protocols. Even now it is advised to prioritize the cases for radiographic examination on appointment basis [9]. The central idea behind restricting the delivery of the treatment was to minimise the human contact and crowding, which in case not maintained can lead to rapid rise in number of COVID-19 cases. At the same time, it should also be born in mind that the health care delivery mechanism should not be crippled as it is a foundation for any prosperous nation. To deal with this situation in a balanced manner, two overlapping concepts were practiced by almost every nation with great success. Firstly, the footfall of patients in the health care unit was restricted by exploring teledentistry. Although, it was not a novel concept, but it was probably not practiced at this large scale. Despite the physical closure, the health care units virtually remained open through teledentistry. The virtual contact was made through telephone, teleconferencing and live chat rooms. Information about them was displayed over the website of the health care centre as well signboards were placed on the walls of physical units of the centre. This facility provided necessary education, guidance, consultation, appointment and referral for the dental care or treatment, hence unnecessary contact was minimised [34].
  The second concept in the wake of minimisminimising human contact which gained wide success was Telephone triaging/teletriaging. It was implemented in many countries with far reaching outcomes [35]. This facility had two-fold benefits as it restricted the number of patients as well as the health care professionals including oral radiology consultants at the health care units. Here the information regarding patient’s health and need for the imaging was assessed [35]. The structured questions during telephone triaging also helped to screen the patient for COVID-19 and render advice accordingly. It also aided in categorising patient’s treatment or imaging need into emergency, urgent or routine care which will be addressed within 1 h, 24 h and 7 days respectively [36]. With this approach, the influx of the patients was regulated and since the radiographic procedures to be carried out per day were reduced, minimal number of radiology technicians will be sufficient at the centre. Similarly, teleradiology will also eliminate the traveling of oral radiologist to the dental clinics. With majority of the radiology setup been digitalized, it is feasible and convenient to share digital images in Jpeg or Tiff format via smart phones or computer devices to the consultant for reporting [37]. For digitalized panoramic and CBCT scans, various software are available for processing DICOM images which will facilitate the practice of teleradiology. When the oral radiologist is working from home platform, the application of “Team viewer” and “Any desk” software will assist consultant to give instructions and command to the console unit installed in the dental radiology centres [37].
- **Reception:** Newer evidence suggests that the act of speech also has a potential to emit tiny droplets that remain dispersed in the environment for a reasonably long duration [29]. Thus, considering in the information back drop, it is advisable for the patients to practice wearing mask at all times including while making a visit to a dental radiology office. Besides, relevant data regarding the patient’s visit, recent travel, contact history and purpose of dental imaging, should gathered by a receptionist who is also wearing a mask while having conversation with patients. The practice of wearing mask will also prevent in disrupting the projectile propagation of the droplets generated during the act of coughing or sneeze [38]. Additionally the reception area can be covered with a physical barrier made up of (acrylic) plexiglass or polycarbonate plastics which are much lighter and better impact resistance than glass. This could be beneficial in cases when the minimal physical safe distancing cannot be maintained during communication [30]. Furthermore, maintaining a reasonable gap between the visits of two patient will enable the personal of dental radiology unit to carry out preventive and disinfection activities of the operatory room [39].
- **Preparation of the staff working in radiology units:** While carrying out the radiographic technique, it is almost inevitable for the personal to establish contact with the patient’s saliva. So considering the risk of transmission of virus, precautions should be considered seriously [40]. Accordingly, HCWs working in dental radiology office including radiographer and radiologist while working in the dental office should use universal personal protective equipment (PPE). The PPE comprises of a protective eyewear, a full cover body gown, mask, head cap and standard vinyl surgical gloves [9]. However, while dealing with known infected cases the standards of PPE must be upgraded such as usage of N95 respirators/filtering face piece (FFP-2)/FFP-3 [13,21,39]. It is essential for the personals working in the dental radiology set-up to undergo training about the infection control protocols, disinfection methods and the disposal of bio-hazardous. The reinforcement of these training sessions is deemed necessary for proper implementation. It is also recommended to have distinct designated places where personals can wear are uncover the protective attire (PPEs) [41].
- **Pre-procedure measures in operatory room:** It is crucial for the procedure room to be duly prepared before starting the imaging. The preparation includes the disinfection of the surfaces which come in constant contact with surface disinfectants such as sodium hypoclocite (NaOCl) in the concentration of 0.1% or 0.5%. Alternatively 70–90% ethanol or 0.5% hydrogen peroxide (H_2_O_2_) can be utilized [10]. Any surface which comes in contact with patients as well as doctors/technicians are considered potential source of contamination, hence all such surfaces needs to covered with plastic barrier films. The surface which comes in that category includes door knobs and parts of operating chair. The portions of the X-ray machine that needs to be protected include buttons of the control panel, X-ray tube head, position indicating device and extendable arms [31]. These plastic films are required to be wrapped prior to the radiographic procedure and taken off immediately after completion of the procedure [42]. Consumables such as face masks, plastic cups and dry face whips should not be left in the procedure room with the fear of getting contaminated while performing procedure. In a situation where conventional radiography is practiced, it is advised to prepare the film and film holder before starting a procedure in a different room [43]. Using a Snap-A-ray film holding device is recommended over the finger technique for stabilizing the film during exposure as the earlier approach will eliminate the risk of salivary contamination (Figure 2). Ideally in a digitalized radiology set-up, the contamination can be restricted by maintaining separate rooms for procedure and image processing (console) [43]. Scientific bodies of Australia, UK, USA and Europe which deals in radiation protection and safety, recommends limited usage of lead apron during oral imaging [44,45]. There is sufficient evidence to support the above recommendation, where the effective dose of radiation absorbed is comparable with or without lead apron, provided the technique has employed high-speed film, rectangular collimation and well optimized equipment. Nevertheless, the usage of lead apron is still in practice with the sole intention to diminish the anxiety and apprehension of the patient, especially for the pregnant women [44,45]. However, it is advisable to use a lead apron in rare imaging technique such as vertex occlusal, where the direction of primary radiation is directed towards radiation susceptible organs. So, it is advisable to keep the lead aprons in the oral radiology centres and should disinfect them with a low level disinfectant such as quaternary ammonium compounds. To prevent contamination, it should not be kept in the exposure room and it should be disinfected immediately after the exposure [10].
- **Ventilation of the operating room:** Generally, radiology offices don’t have windows as the room is completely sealed off for effective air conditioner. Hence it is suggested to devise a mechanism wherein the air inside the room is expelled out via a high efficiency particulate arrestance (HEPA) filter which has an efficiency to filter the suspended particles measuring size of 0.3 microns [46]. This modification will be beneficial in reducing the risk of personals from getting infection [47]. Instances where these changes are not practically feasible, it is advised to restrict intraoral imaging as these will add more quantity of aerosols from the act of cough or gag reflex [48]. Digital orthopantomography (OPG) and cone beam computed tomography (CBCT) scanning can be used as an alternative, considering the availability and permissible level of radiation exposure. In an attempt to minimise the radiation exposure, width of image and field of view (FOV) can be appropriately used in OPG and CBCT respectively [10,49]. However, if intra-oral radiographs are absolutely unavoidable, measures should be taken to minimise the risk of aerosols. The series of precautions include conditioning of patient by providing prior explanation of the entire procedure. Patient should also be told to abstain from conversation and coughing during the procedure [50]. A pre-procedural mouth rinse containing 1% H_2_O_2_ for 30 s or 0.2% povidine iodine solution has a potential to reduce the viral count [10]. Besides, proper projection technique should be adopted to avoid delay and repeat radiographs. Installation of exhaust fans in the procedure rooms can be the most simplistic modification that can be made to release the potentially infected air outside. Further studies are warranted in these directions to explore the present hypothesis.
- **Decontamination of oral radiology clinics:** There are several ways to decontaminate the operatory which are been practiced in different health care settings for the prevention of nosocomial infection. However, there efficacy in combating SARS-CoV-2 needs further research.
  1. Fogging: It is a process wherein the liquid is converted aerosols which has small particle and appears as mist. This can be achieved by chemicals using H_2_O_2_, AgNO_3_ or NaOCl [51]. This method is already practiced for the decontamination of Operation Theatre [52]. However, its usage in oral radiology clinics is questionable as it might tamper the wiring of panoramic and cone beam machine [53].
  2. Ozonation of the air: Ozone (O_3_) has proven its disinfection potential against various microbes including viruses. It has been used for various purposes such as therapeutic and disinfection. The gaseous form of O_3_ is commonly used for disinfection. James Hudson et al. devised a machine which was designed to generate O_3_, which will later covert into oxygen upon dissociation to bring about disinfection [54]. Fortunately, O_3_ gas has not shown any detrimental effect on the equipment and devices in the oral radiology unit. Nevertheless, its potential usage for COVID-19 is yet to be determined.
  3. Ultraviolet light C: In a study the ultraviolet C (100–280 nm) light has shown the maximum potential to disinfect the ICU room. The point of consideration during this process is the distance from the UV light and the target area. Additionally, the objects should be clean prior to ultraviolet C radiation as organic material can absorb the radiation and block the reflection. It can be potentially hazardous if not used properly as per the protocol [55]. Currently, the usage of UV-C on SARS CoV-2 has not been estimated.
- **Record room:** Conventionally the patient’s case files, radiographs or any other related documents are either stored in the operatory room or separate room. With the inception of digitalized radiography, it is easier to maintain paperless documentation. Although, some institutions/clinics are still practising paper. It is preferable to have a separate room outside the operatory room for the purpose of storing dental records [56].
- **Storage and strategic stockpiling:** Researchers have stated that SARS CoV-2 can remain alive on environmental surfaces varying from few hours to days [57]. Various parameters such as room temperature and moisture content of the room plays important role in life expectancy of the SARS CoV-2. The dental radiology stock including radiographs and processing solutions are stored in an air-tight plastic container, which makes them easy for disinfection and storage. It has been reported that SARS CoV-2 can stay viable on plastic containers for up to three days, therefore it is advised to abstain from touching for the same number of days [56]. The consumption of stock should follow the concept of first-in, first-out, (FIFO), wherein the oldest stock is taken out first for consumption. In order to maintain so, the arrangement of stock should be made strategically for the easy movement of old and new stock. Care should be taken to avoid over-stocking by planning the periodic purchases considering the consumption and quantity of available stock.
- **Specific guidelines for different dental imaging modalities:** The guidelines will vary as per the scale of radiology set-up (small vs. large), nature of setting (academic vs. clinical), availability of the equipment (intraoral vs. extraoral) and mode of imaging practiced (film based vs. Digital). Although the basic infection control protocols stand applicable irrespective of variation in set-up, but few modifications are specific to the dental imaging modality. The following section provides a list of recommendations based on the imaging modality used in the radiology set-up.
  1. Intraoral imaging—With the maximum possibility of transmission of disease and cross contamination with the involved salivary exposure, it has become the least preferred imaging option during this pandemic [57]. Intraoral imaging can induce hypersalivation, vomiting or coughing which can be a potential source of infection [21]. However, it’s wider availability, high resolution images and less radiation exposure made it the fundamental imaging option in pre pandemic era [33]. Even today, its diagnostic importance cannot be denied. Hence, under strict preventive measures it can be practiced. The common preventive measures for disinfection practiced before and after the procedure irrespective of imaging modality remains the same. Films/sensors wrapped with a double barrier are used along with film holders wrapped with barriers too (Figure 2). In case of digital set-up, for providing additional protection, sensors can have an outermost wrapping of disposable latex finger cot [10]. Even the cable of wired sensors needs to be wrapped with barrier films. Charge coupled device and phosphor storage plates need to be wiped with 70% alcohol-based disinfectant or ethylene oxide [57]. The most sensitive activity in intraoral imaging is the handling of exposed films from the operatory room to the dark room. “No touch” technique has to be strictly adopted for this purpose [10]. Patients with serious gag-reflex needs to be managed before taking intraoral radiograph [10,56]. Alternatively, occlusal radiographs can be considered. In general, the digital imaging and teleradiology has emerged as the need of the hour and needs to be implemented at the earliest. In a small scale dental clinical setup, where an isolated radiology setup is not feasible, it is advisable to use a hand-held dental radiology system can be used in compliance with current infection control protocol.
  2. Panoramic imaging–During this pandemic, panoramic imaging has emerged as the most preferred imaging modality as the probability of saliva contamination is minimal [56]. Despite being popular, demerits associated with this imaging technique such as metallic artifact, compromised resolution, and magnification of the image cannot be ignored. Based on the area of interest and the amount of details required, sectional or full width panoramic image can be taken. In order to prevent cross contamination, portions of equipment which come in contact need to be wrapped with barrier film [10,57]. The preparation of machine such as loading the cassette and adjusting the image settings should be done beforehand. The patient preparation in terms of removing the metallic objects needs to be completed before he/she enters the operatory room. As per the new protocol, patients should be encouraged to wear fresh piece of mask and gloves during the exposure [56]. Instead of bite peg and patient positioning device, an alternative approach should be adopted [10,56]. Anatomical landmarks such as lip commissure, ala-tragus line and indicating beam can be used for the positioning and alignment of correct posture. Two operators are suggested to divide the activities during the process as this will expedite the process and also eliminate the chances of cross-contamination [56]. Lastly, the disinfection of the room, changing the barrier films and, maintaining a reasonable gap between the two patients (˃30 min) will be equally crucial in efforts made towards preventing cross contamination [56].
  3. CBCT—Besides the general infection control measures, the portions of machine which comes in repeated contact of patient or radiographer needs to be covered with barrier film [10]. Aids available for aligning patients can be avoided; instead position indicator lights can be taken as guide for the purpose (Figure 3A,B). Importantly, the bite pegs should have disposable barrier for every patient and its usage should be completely avoided while dealing with an infected patient.
  4. Ultrasonography (USG)—In the recent years, ultrasound imaging has gained popularity in oral and maxillofacial radiology while assessing the head and neck pathologies. Its non-invasive nature, real time imaging and non-usage of ionizing radiation have contributed towards wider usage in head and imaging especially for major salivary gland tumours and lymphadenopathy [58].
    The transducer used for imaging is known to nurture microbes as it remains in contact with the tissues. The intraoral transducers carry even higher risk of harbouring microorganism and thus considered as potential source of contamination. Keeping this in view, the European Society of Radiology Ultrasound Working Group, has laid robust measures to minimise the risk of cross-contamination [10,59]. The recommendations include proper protocol for cleaning and disinfection using quaternary ammonium compound disinfectants. Transducers that carry higher risk should be disinfected using UV light and chemical disinfection. In addition to transducers, other components such as console, keyboards or cables of ultrasound needs either to be disinfected or covered with barrier films which should be changed frequently. Strict compliance with the hand hygiene measure is deemed necessary [59].

### 3.5. Dental Radiology Set-Up in Academic Institutes

With the forced implementation of isolation and social distancing, the traditional method of teaching and training was enormously affected. It leads to an upsurge in distance learning as the mainstream mode of teaching. With regard to the theoretical segment of the dental radiology curriculum, it was carried out in virtual classrooms with the help of various web-based forums [60]. Despite the benefits of virtual session such as accessibility to remote areas and flexibility in scheduling the classes, there are few consequential concerns among professors, students as well as parents. However, the practical component, especially the radiographic techniques and patient care while taking radiographic projections cannot be justified in the same way. The intraoral radiographic techniques have an indispensible significance in curriculum. Since, they are associated with high risk of contamination [61]; institutes should encourage extraoral projection modalities [62]. In view of limitations in panoramic or CBCT equipment, strict infection control measures should be enforced while practising intraoral techniques. The measures advised include usage of extra barrier for films/receptor, film holders and PPEs while working in the clinical setting [63,64]. Additionally, switching to digital imaging will fetch benefits of tele-radiology and also eliminates the risk of cross contamination [65,66]. Various institutions have adopted novel techniques such as interactive case discussion over pinterest^®^ to deal with the situation [67]. Past researches have shown promising results with E-learning in interpretation exercises over didactic learning [68].

In the view of predictions with regard to new surge in COVID-19, the present restriction seems to be continued for coming few years [69]. Hence, there is a need of modification in the curriculum as well as the approach in delivering practical session in oral radiology, specifically, updating in the infection control protocols [64].

## 4. Conclusions

Saliva and aerosol are closely related to dentistry and an established route of disease transmission. Among the dental radiographic imaging modalities, the highest risk of cross contamination is associated with intraoral radiographic techniques. Hence, extraoral techniques such as panoramic and CBCT are preferred with a caution for radiation exposure. Thus currently, in the pandemic of COVID-19, strict adherence to infection control protocol is the only way to decelerate the disease transmission. Academic institutes also need to adopt similar measures in their setting in addition to modification in the curriculum so as to meet the exceptional circumstances which are expected to stay with us for some years.
